# Relationship between polymorphisms in beta -2 adrenergic receptor gene and ischemic stroke in North Indian Population: a hospital based case control study

**DOI:** 10.1186/1756-0500-7-396

**Published:** 2014-06-25

**Authors:** Amit Kumar, Manjari Tripathi, Madakasira Vasantha Padma Srivastava, Subbiah Vivekanandhan, Kameshwar Prasad

**Affiliations:** 1 Department of Neurology, Room No. 704, Neurosciences Centre, All India Institute of Medical Sciences, Ansari Nagar, New Delhi, India; 2 Department of Neurobiochemistry, All India Institute of Medical Sciences, New Delhi, India

## Abstract

**Background:**

Stroke is a multi-factorial disease and influenced by both genetic and environmental factors. The purpose of the present case control study was to check the relationship between beta-2 adrenergic receptor (ADRB2) polymorphism and ischemic stroke in North Indian Population.

**Methods:**

In a hospital based case control study, patients with ischemic stroke and control subjects from outpatient department and neurology ward of All India Institute of Medical Sciences New Delhi. Genotyping was performed by using Polymerase chain reaction–Restriction fragment length polymorphism. Frequency distributions of genotypes and alleles were compared between cases and controls using multivariate logistic regression.

**Results:**

In this study, 224 patients and 224 age-and sex-matched control subjects were recruited. Mean age of cases and controls were 53.9 ± 13.4 and 53.6 ± 12.9 years respectively. Multivariate logistic regression analysis showed an independent association between Gln27Glu polymorphism and large vessel stroke (LVD) under a recessive model of inheritance (OR 3.9; 95% CI 1.3 to 11). An age-stratified analysis, suggested independent association between Gln27Glu polymorphism and ischemic stroke, large vessel disease and small vessel disease stroke who had onset of disease at an older age.

**Conclusions:**

The findings of the present study suggest that Gln27Glu polymorphism of the ADRB2 gene may confer higher risk of large vessel disease stroke in a North Indian population. Prospective studies with larger sample size are required for independent validation.

## Background

Stroke is the second most common cause of death after ischemic heart disease [1], but, it ranks first in the Asian regions that have the highest population in the world [2]. There are 16.9 million incident stroke cases worldwide, twice as many stroke survivors, and 5.9 million deaths from stroke [2]. Incidence of stroke in South Asian countries have increased by more than 100% while this is deceased by 42% in developed European courtiers in last four decade [3]. World Health Organisation estimates suggest that by 2050, 80 percent of stroke cases in the world would occur in low and middle income countries, mainly India and China [4].

**Figure 1:**
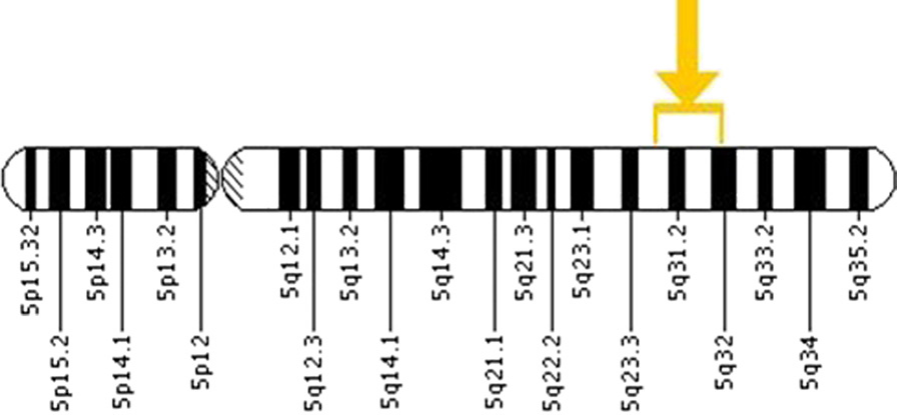
Location of beta-2 receptor in chromosome 5q31–32.

**Figure 2:**
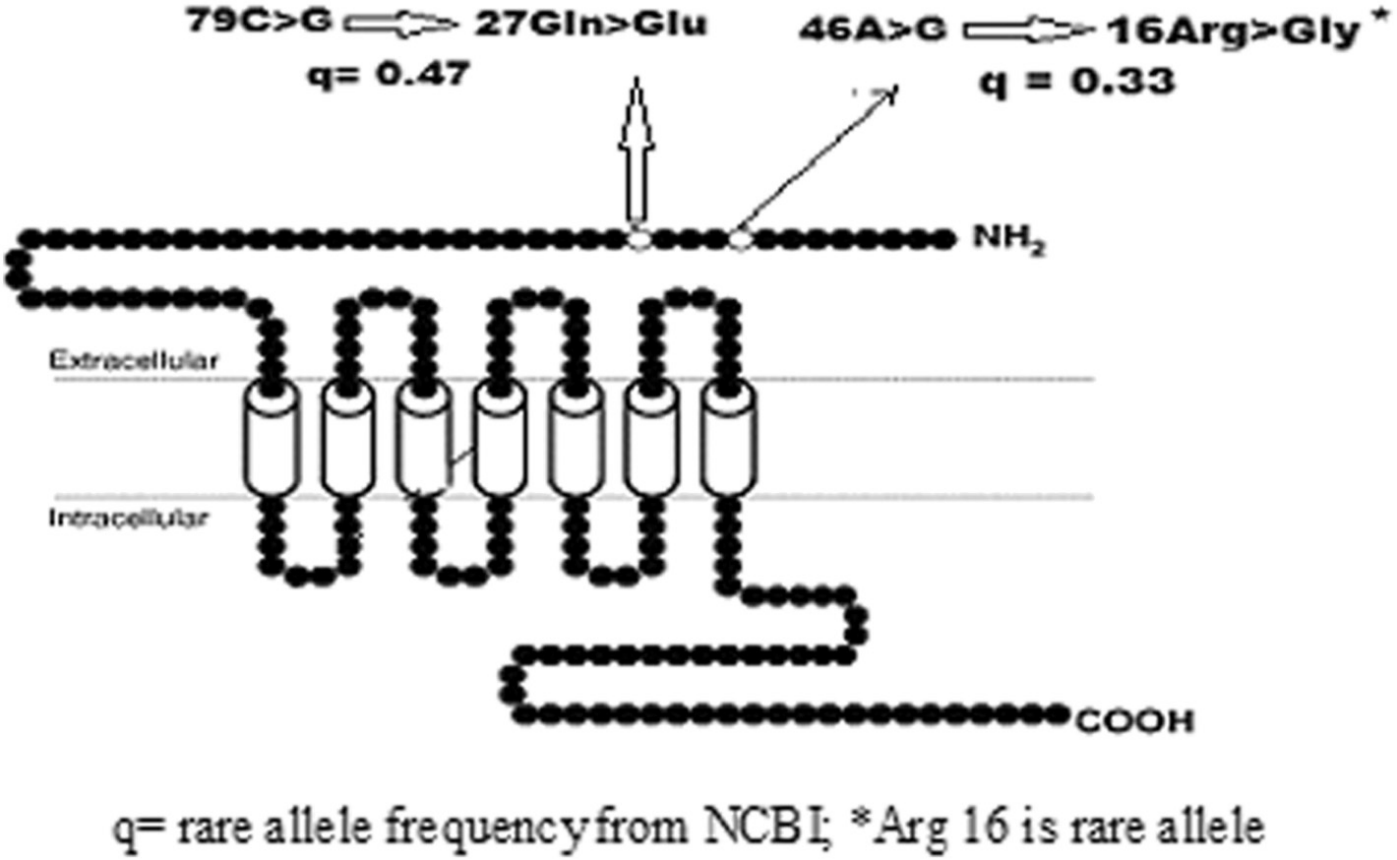
Localization and characterization beta-2, adrenergic receptor polymorphism studied.

Stroke is a multifactorial, polygenic disorder, influenced by both environmental and genetic factors. There are several risk factors has been discovered for stroke, such as hypertension, diabetes, smoking, dyslipidemia etc., however these risk factors do not explain why some individual are more susceptible to these environmental determinants in comparison to others with same given risk factors [5]. A growing body of evidence, suggesting that genetic variant may predispose to developing stroke [6]. The beta-2 adrenergic receptor, coded by an intronless gene on the long arm of chromosome 5q31–32 (Figure 1) and found in multiple tissues (Table 1). It is members of the superfamily of G protein-coupled receptors that generally modulate action of catecholamine by an increase in level of secondary messenger cyclic AMP. It has been shown that agonist-independent, G-protein coupled receptor activity regulates anterior-posterior targeting of olfactory sensory neurons [7]. Reduced expression of beta-2 adrenergic receptor contributes to the age- related deterioration of glucose tolerance [8]. It is broadly recognized that the beta-blocker therapy shows better response in younger patients than hospitalized senescent patients [9]. Cathecolamine-induced adenylate cyclase activity has been shown to decrease during ageing in human lymphocytes [10,11]. The age-related deterioration in beta-adrenergic receptor function and subsequent cyclic AMP generation [12] that is a common factor underlying atherosclerosis, vascular insufficiency and hypertension [13] and these mechanisms are believed to confer increased risk for cardiovascular and cerebrovascular diseases [14]. Cyclic AMP is also a possible target for prevention and treatment of atherosclerosis [15]. It is reported in the recent year that these polymorphisms are related to many kinds of clinical diseases such as asthma, hypertension, diabetes, heart diseases [16]. In the present study, we examined the relationship of Arg16Gly and Gln27Glu polymorphisms of beta-2 adrenergic receptor (Figure 2) as a risk factor for ischemic stroke in the North India in order to facilitate early diagnosis of the susceptible population and prevention of ischemic stroke.

**Table 1:** Distribution and functions of beta adrenergic receptor

**Subtype**	**Tissue distribution**	**Functions**	**Gene localization**	**Common functional variants**
BAR-2	Lung, Brochial	Bronchodilation	5q31-q32	Arg16Gly
	Vascular smooth muscle	Vasodialation		Gln27Glu
	Heart	Positively inotropic and chronotropic		
	Uterus	Relaxation		
	Bladder	Relaxation		
	Adipocytes	Lipolysis		
	Eye	Increase aqueous humor		
	Liver	Glycogenolysis		
	Skeletal Muscle	Glycogenolysis		
	Sympathetic terminal	Nor-epinephrine release		

*Abbreviation*: *BAR* beta adrenergic receptor.

## Methods

During this study, 224 patients with ischemic stroke and 224 control subjects were enrolled from the ward and outpatient department of Neurology, All India Institute of Medical Sciences (AIIMS), New Delhi, India from the period of February 2010 to April 2012. Ischemic stroke was defined as rapidly developing clinical signs of focal (or global) disturbance of cerebral function, with symptoms lasting more than 24 hours or longer or leading to death, with no apparent cause other than of vascular origin [17]. Stroke is a heterogeneous phenotype, therefore, analysis based on specific stroke cause in important in genetic association studies, therefore, we used TOAST criteria to determine stroke aetiology [18], which include large vessel stroke which occurs usually due to atherosclerosis or occlusion of major brain artery or branch cortical artery and small vessel stroke which occur due to the involvement of small perforating end arteries within the brain. All subjects underwent standardized clinical evaluations. The study was approved by the All India Institute of Medical Sciences, New Delhi ethics committee (Ref No. P-02/06.10.2008). Informed consent was obtained from each patient/control subject or from the relatives or a legal representative in the case of critically disabled patients. Inclusion and exclusion criteria for cases and control subjects are same as earlier published article by our team [19]. Definitions of variables are same as in the published study [5].

### Sample size

Sample size calculation was based on parameters of the study reported by Stanzione *et al.*[20], on beta-adrenergic receptor polymorphism in ischemic stroke in which prevalence for the beta-adrenergic receptor variant Glu27Glu genotype was 0.32 in control group and odds ratio for this polymorphism was 1.68. Assuming 80% power and 5% alpha, with one control per case, we obtained estimated minimum sample size were 212. 224 patients and 224 controls were included in this study to compensate for any loss of sample.

#### *Genomic DNA extraction*

Genomic DNA was isolated by conventional Phenol Chloroform Method. DNA concentrations were estimated by measuring the absorbance at 260 and 280 nm using a nanodrop spectrophotometer (Gene Quant, Pharmacia, USA) and its quality was assessed by the A260/A280 ratio. DNA samples with a 260/280 ratio ranging from 1.5 to 1.8 were processed for PCR amplification.

#### *Detection of gene polymorphisms*

Genotyping: Primers used for the amplification of Arg16Gly polymorphism of beta-2 adrenergic receptor is: forward: 5’-CTTCTTGCTGGCACGCAAT -3’ reverse: 5’-CCAGTGAAGTGATGAAGTAGTTGG -3’, (PCR product length was 201 bp, restriction enzyme BsrD1; Ferments; 2.5 U for 6 h at 37°C). For determination of Gln27Glu polymorphism, primer used were forward 5’-GAATGAGGCTTCCAGGCGTC-3’ reverse 5’- GGCCCATGACCAGATCAGCA-3’ (PCR product length was 356 bp; restriction enzyme FNu4HI Fermentas; 0.5 U for 6 h at 37°C). The restriction products were separated by electrophoresis in 2.5% agarose gels and visualized in UV light. Gel Images were documented by using gel doc.

### Statistical analyses

Allele frequencies were compared using the Chi square test. The conditional logistic regression model was used to estimate Odds ratio (OR) and 95% confidence intervals (CIs) for association of beta-1 receptor polymorphisms with ischemic stroke. Multivariate logistic regression was used to control the confounding effect of demographic and risk factor variables. Tests were considered significant at *P* < 0.05. The chi square test was used to determine the frequency distribution of genotypes, were in accordance with Hardy-Weinberg equilibrium (HWE). Data were analyzed using the SPSS statistical package, version 17.0 (SPSS, Chicago, IL, USA).

## Results

Demographic and risk factor variables in cases and controls are given in (Table 2). Multivariate analysis did not show a significant association between Arg16Gly polymorphism and ischemic stroke (Table 3 and Additional file 1: Table S1). Analysis stratified by hypertension and age showed weak associations of Arg16Gly polymorphism among some of subgroups (Additional file 1: Table S2). Borderline significance was observed between Gln27Glu polymorphism of beta-2 AR and ischemic stroke (OR, 2.8; 95% CI, 0.98 to 8.1; *P* = 0.05) under recessive model of inheritance (Table 3). Distribution of genotypic and allelic frequencies of polymorphism at Gln27Glu (SNP 79 C>G) position of beta-2 adrenergic receptor gene in controls and ischemic stroke and its subtypes are given in Additional file 1: Table S3. Significant association was observed between Gln27Glu polymorphism and LVD stroke (OR, 3.9; 95% CI, 1.3 to 11; *P* = 0.01) in adjusted analysis under the recessive model of inheritance (Table 3). Age stratified analysis, showed an independent significant association between Gln27Glu variant and risk of overall ischemic stroke, *LVD* stroke and SVD stroke, assuming both recessive and dominant model of inheritance only in the patients, those who had late onset (>50 years) of disease (Additional file 1: Table S4). Individuals carrying haplotype Arg16-Glu27 were found to be 4.5 fold higher risk of ischemic stroke as compared to control (OR, 4.5; 95% CI, 2.9 to 7). Gly16-Glu27 haplotype was significantly associated with risk of ischemic stroke (OR, 2.3; 95% CI, 1.7 to 3.2) (Table 4).

**Table 2:** Demographic and risk factors variables of Ischemic stroke patients and controls

**S.No**	**Variables**	**Case**	**Control**	**Unadjusted OR (95% CI), p value**	*Adjusted OR (95% CI), p value
1	^a^Age ± SD (years)	53.9 ± 3.4	53.6 ± 12.9	Matched	Matched
2	Male/Female	183/41	183/41	Matched	Matched
3	Hypertension n (%)	138 (62%)	48 (21%)	5.8 (3.8 to 8.8), < 0.001	6.6 (3.4 to 12.9), <0.001
4	Smoking n (%)	98 (44%)	53 (24%)	2.4 (1.6 to 3.6), < 0.001	2.8 (1.5 to 5.2), 0.001
5	Diabetes n (%)	67 (31%)	35 (16%)	2.4 (1.5 to 3.8), 0.001	1.5 (0.85 to 2.8), 0.15
6	Migraine n (%)	20 (9%)	15 (6%)	1.3 (0.67 to 2.8), 0.37,	NA
7	Dyslipidemia n (%)	96 (43%)	37 (17%)	3.6 (2.3 to 5.7), < 0.001	3.8 (1.9 to 7.7), <0.001
8	FHS n (%)	48(21.5)	15(6.9)	3.7 (2 to 6.8), < 0.001	5.2 (2.3 to 11.5), <0.001

^a^Age of stroke patients at the time of onset; SD, standard deviation; FHS, family history of stroke; NA, Not applicable (Excluded from the model as statistically not significant).

**Table 3:** Association between Arg16Gly and Gln27Glu polymorphisms of beta -2 adrenergic receptor and Ischemic stroke

**Polymorphism**	**N**	**Sample**	**Genotype distribution (%)**			**HWE**	**Recessive**	**Dominant**	**Sample**	**Allele distribution (%)**		**OR [95% CI],**
						**P value**	**Adjusted**	**Adjusted**				**P value**
							**OR [95% CI],**	**OR [95% CI],**				
**Arg16Gly**			**Arg/Arg**	**Arg/Gly**	**Gly/Gly**		**P value**	**P value**		**Arg**	**Gly**	
	224	IS	21	53	26	0.39	1.5	1.5	IS	47	53	1.3 [0.7 to 2.3],
	224	Controls	28	52	20	0.43	[0.76 to 3.2],	[0.74 to 3.3],	Control	54	46	0.32
							0.21	0.23				
	76	LVD	20	55	25	0.34	1.2	0.97	LVD	47	53	1.3 [0.7 to 2.3],
	224	Control	28	52	20	0.43	[0.21 to 7.6,]	[0.07 to 13],	Control	54	46	0.32
							0.78	0.98				
	78	SVD	19	50	31	0.88	2.9	2.4	SVD	44	56	1.4 [0.8 to 2.6],
	224	Control	28	52	20	0.43	[0.71 to 12],	[0.43 to 13],	Control	54	46	0.15
							0.13	0.31				
**Gln27Glu**			**Gln/Gln**	**Gln/Glu**	**Glu/Glu**					**Gln**	**Glu**	
	224	IS	44	40	16	0.07	2.8	1.5	IS	64	36	1.6 [0.91 to 3.1],
	224	Controls	55	39	6	0.59	[0.98 to 8.1],	[0.91 to 2.7],	Control	75	25	0.09
							0.05	0.10				
	76	LVD	47	35	18	0.04	3.9	1.5	LVD	64	36	1.6 [0.91 to 3.1],
	224	Control	55	39	6	0.59	[1.3 to 11],	[0.81 to 2.9],	Control	75	25	0.09
							0.01	0.18				
	78	SVD	42	45	13	0.87	0.97	1.4	SVD	65	35	1.6 [ 0.87 to 2.9],
	224	Control	55	39	6	0.59	[0.15 to 6.1]	[0.44 to 5],	Control	75	25	0.12
							0.98	0.51				

*Abbreviations*: *IS* ischemic stroke, *OR* odds ratio, *CI* confidence interval, *LVD* large vessel stroke, *SVD* small vessel stroke.

**Table 4:** Haplotype frequencies of beta-2 adrenergic receptor Arg16Gly and Gln27Glu polymorphisms

**Haplotype**	**Case n (%)**	**Control n (%)**	**OR (95% CI)**	***P *** **Value**
Arg16-Gln27	115 (25.7)	205 (45.7)	Reference	
Arg16-Glu27	97 (21.5)	38 (8.5)	4.5 (2.9 to 7)	<0.001
Gly16-Glu27	172 (38.4)	129 (28.8)	2.3 (1.7 to 3.2)	<0.001
Gly16-Gln27	64 (14.4)	76 (17)	1.5 (1.001 to 2.2)	0.04
Total	448	448		

*Abbreviations: OR* odds ratio, *CI* 95% confidence interval, *Gly* Glycine, *Arg* Arginine, *Glu* Glutamic acid, *Gln* Glutamine.

## Discussion

In this hospital based case control study, a common functional polymorphism at Gln27Glu position of beta-2 AR was found to be associated with more than threefold increased risk of LVD stroke.

The present study is the first study, up to the best of our knowledge, showing the association of Arg16Gly and Gln27Glu polymorphisms of beta-2 adrenergic receptor with ischemic stroke in Asian population. Frequency distribution of variant alleles of Arg16Gly and Gln27Glu polymorphisms of beta-2 adrenergic receptors in our study are consistent with the other previously reported studies (Table 5).

**Table 5:** Frequency distribution of variant allele of Arg16Gly and Gln27Glu polymorphism in few of different ethnic regions

**Study design**	**Year**	**Allele frequency (%)**		**Population**	**Sample size**	**Condition**	**Reference**
		**Gly16**	**Glu27**		**Case/control**		
		**Case/control**	**Case/control**				
Case control	1998	NA	0.43/0.36	Denmark	156/205	Obesity	[21]
Case control	2006	NA	0.26/0.15	Saudi Arabia	773/528	Coronary artery disease	[22]
Case control	2007	0.66/0.65	0.57/0.48	Italy	294/286	Stroke	[20]
Case control	2011	0.46/0.43	0.17/0.14	Chinese	238/265	Asthma	[23]
Case control	2012	0.49/0.39	0.07/0.12	Malaysia	50/50	Hypertension	[24]
Case control	2005	0.40/0.38	0.43/0.41	California	194/819	Myocardial infarction	[25]
Cohort study	2009	0.58	0.29	Brazil	501	Heart Failure	[26]
Meta-analysis	2012	0.47/0.46	0.48/0.17	Asian	1097/844	Chronic obstructive pulmonary disease	[27]
Meta-analysis	2012	0.61/0.62	0.41/0.40	Caucasian	727/850	Chronic obstructive pulmonary disease	[27]
Case control	2012	0.60/0.65	0.27/0.30	North Indian	410/414	Asthma	[28]
Case control	2014	0.52/0.58	0.47/0.23	North Indian	106/106	Hemorrhagic Stroke	[29]
Present study	2014	0.53/0.46	0.36/0.25	North Indian	224/224	Ischemic Stroke	Present study

In vitro studies have shown higher agonist induced desensitization for Gly16 variant in comparison to Arg16 variant after the administration of isoprenaline which is a nonselective beta adrenergic agonist [30]. In vitro study [31] has indicated that Glu27 variant produces a higher degree of responsiveness to adrenergic agonist with resistance to down regulation relative to wild type allele, however, strong linkage disequilibrium between codon 16 and codon 27 and there is greater probability that subjects who are carrying Glu27Glu genotype are mostly homozygous for Gly16 allele than Arg16 [32]. Our study also showed similar linkage disequilibrium. Gly16-Glu27 haplotype showed enhanced receptor down regulation after exposure to isoprenaline when compared with Arg16-Glu27 [30]. Due to strong linkage disequilibrium, it has been suggested that subject carrying Gly16-Glu27 haplotype may have greater down regulation and desensitization as compared to Arg16 allele [30,33]. Down regulation of this receptor may lead to decrease in concentration of cyclic AMP in the vascular smooth muscle, lungs, kidney, and sympathetic terminal. This decrease in cyclic AMP leads to modulation of atherosclerosis, recruitment of reactive oxygen species, and recruitment of circulating monocytes to the artery wall and their differentiation into macrophage foam cell, by controlling the expression of proinflammatory and anti-inflammatory interleukin [15]. By these mechanisms, beta-2 adrenergic receptor gene polymorphisms might influence the genetic predisposition to ischemic stroke. Several studies have shown the independent association of Gln27Glu polymorphism of beta-2 adrenergic receptor gene polymorphism with a number of diseases like obesity [34,35], dyslipidemia [36], myocardial infarction [16], diabetes [37]. Few studies have addressed the association of beta-2 receptor polymorphism with ischemic stroke and both positive and negative associations have been reported. A case control study, reported from Italy, indicated a positive association between Gln27Glu polymorphism and Ischemic stroke [20]. A previous study by Heckbert et al., failed to show significant association of beta-2 adrenergic receptor variant with the incidence of stroke [38]. A prospective cohort study included 25225 women showed that the different haplotypic combination of beta receptor gene variant did not affect the incidence of ischemic stroke in women [39]. A case control study reported by Zhao *et al.* did not find significant association of Gln27Glu polymorphism with risk of ischemic stroke, however, higher frequency of variant Glu27 allele in ischemic stroke patients (0.11) than controls (0.07) has been observed [40]. In all the published case control studies (*three*), the frequency of the risk allele (Glu27) was higher in cases as compared to controls. Our study also indicated a significant association between Gln27Glu polymorphism and ischemic stroke associated with large vessel disease.

Hypertension is a most important and common risk factor for stroke. A meta-analysis published by Lou Y, et *al.,*[41] showed significant association between Arg16Gly polymorphism and risk of hypertension under a dominant model in a subgroup of mixed African (OR, 1.18, 95% CI, 1.01 to 1.87, P = 0.04). This meta-analysis also indicated the significant association between Gln27Glu polymorphism and hypertension under a dominant model of inheritance (OR, 1.38; 95% CI, 1.02 to 1.86) [41]. Other polymorphisms such as on gene CaMK4 (calcium/calmodulin-dependent kinase 4) which has a role in regulation of vascular tone also have been shown to be associated with hypertension [42]. A previous study has demonstrated that more than 85% patients with CHD have at least 1 of five modifiable risk factors (hypertension, diabetes, and hypercholesterolemia, smoking and family history) [43,44]. A previous study indicated that the P (A2) polymorphism is a genetic determinant of ischemic stroke in a high-risk hypertensive population [45]. Our analysis, stratified by hypertension indicates that Glu27 variant is associated with both the hypertensive and non-hypertensive stroke subjects and shows a similar pattern of associations in both the groups (Additional file 1: Table S4).

The age-related decline in beta-adrenergic receptor function and subsequent cyclic AMP generation [12] is a common factor underlying atherosclerosis, vascular insufficiency and hypertension [13]. A study has shown that age-associated decline in beta-adrenergic receptor sensitivity in the cardiovascular system [46]. Our age-stratified analysis, showed significant associations of Gln27Glu polymorphism in only those patients who had onset of disease at late (>50 years) stage. During old age conventional risk factors of stroke becomes more prevalent and it is possible that Glu27 variant may interact at later stages of age with other conventional risk factors that cause stroke.

In the present study, individuals who had Arg16-Glu27 haplotype were found to have a higher risk of ischemic stroke. This association shows the additive effects of both the alleles and may have enhanced combined impact of ischemic stroke risk. The present study suggests that Haplotype Gly16-Glu27 was most prevalent haplotype and significantly associated with risk of ischemic stroke. This is consistent with finding of the study reported by Stanzione *et al.*[20].

Studies have shown that there is a relationship between the effectiveness of beta-blocker treatment and beta adrenergic receptor polymorphism [47]. Further studies are required to check the significance of beta blocker treatment and stroke prevention in carrier of beta-adrenergic receptor polymorphism.

### Limitation of the study

The sample size of the present study might not be large enough to detect small effects of low penetrance SNPs. Each susceptible polymorphism may only contribute to mild effect, thus the analysis of single SNP could be ambiguous by unstudied polymorphisms and environmental factors that influences the phenotype. Borderline deviation from HWE was observed in the distribution of genotypes in patients with LVD stroke. We cannot completely exclude the effects of residual confounding attributable to the measurement error in the assessment of confounding factors. In the present study, the numbers of male patients were more than double the number of female patients, which substantiates that results of this study may be applied to male ischemic stroke populations.

## Conclusion

In conclusion, this study provides evidence that the presence of Arg16-Glu27 haplotype of beta-2 adrenergic receptor in the subjects might confer increased risk of developing ischemic stroke. Gln27Glu polymorphism may confer increased risk for developing stroke associated with large vessel. Identification of a subgroup of patients, who are carrier of beta-adrenergic receptor polymorphism, may have implication for planning their future treatment strategies. Prospective studies with larger sample size are required for independent validation.

## Competing interests

The authors declare that they have no competing interests.

## Authors’ contributions

AK has participated in the writing of manuscript, conception, design and conduct of the study. KP has participated in the design, analysis and interpretation of data. MT, MVPS and SV have participated in the conception and design of the study. All authors read and approved the final manuscript.

## Supplementary Material

Additional file 1 **Table S1.** Distribution of genotypic and allelic frequencies of polymorphism at Arg16Gly (SNP 46 A>G) position of beta-2 adrenergic receptor gene in controls and ischemic stroke and its subtypes. **Table S2.** Analysis stratified by hypertension and age for association of polymorphism at Arg16Gly (SNP 46 A>G) position of beta-2 adrenergic receptor gene with ischemic stroke and its subtypes. **Table S3.** Distribution of genotypic and allelic frequencies of polymorphism at Gln27Glu (SNP 79 C>G) position of beta-2 adrenergic receptor gene in controls and ischemic stroke and its subtypes. **Table S4.** Analysis stratified by hypertension and age for association of polymorphism at Gln27Glu (SNP79C>G) position of beta-2 adrenergic receptor gene with ischemic stroke and its subtypes. Click here for file
