# Supplementary material for: Relationship between polymorphisms in beta -2 adrenergic receptor gene and ischemic stroke in North Indian Population: a hospital based case control study
Source: BMC Res Notes. 2014 Jun 25;7:396. doi: 10.1186/1756-0500-7-396 (PMC4091742; doi:10.1186/1756-0500-7-396)
Supplement: Additional file 1 — Table S1. Distribution of genotypic and allelic frequencies of polymorphism at Arg16Gly (SNP 46 A>G) position of beta-2 adrenergic receptor gene in controls and ischemic stroke and its subtypes. Table S2. Analysis stratified by hypertension and age for association of polymorphism at Arg16Gly (SNP 46 A>G) position of beta-2 adrenergic receptor gene with ischemic stroke and its subtypes. Table S3. Distribution of genotypic and allelic frequencies of polymorphism at Gln27Glu (SNP 79 C>G) position of beta-2 adrenergic receptor gene in controls and ischemic stroke and its subtypes. Table S4. Analysis stratified by hypertension and age for association of polymorphism at Gln27Glu (SNP79C>G) position of beta-2 adrenergic receptor gene with ischemic stroke and its subtypes. [file 1756-0500-7-396-S1.doc]

**Table S1.** Distribution of genotypic and allelic frequencies of polymorphism at **Arg16Gly**(SNP 46 A>G) position of beta-2 adrenergic receptor gene in controls and ischemic stroke and its subtypes

| **Group** | **No** | **Genotypes** | | | **(Allele) Gly vs. Arg** | | | |
| --- | --- | --- | --- | --- | --- | --- | --- | --- |
|  |  | Arg/Arg | Arg/Gly | Gly/Gly | Arg:Gly | OR | CI | P |
| Con | 224 | 63 (28%) | 117 (52%) | 44 (20%) | 0.54:0.46 |  | | |
| IS | 224 | 47 (21%) | 118 (53%) | 59 (26%) | 0.47:0.53 | 1.3 | 0.7 to 2.3 | 0.32 |
| LVD | 76 | 15 (20%) | 42 (55%) | 19 (25%) | 0.47:0.53 | 1.3 | 0.7 to 2.3 | 0.32 |
| SVD | 78 | 15 (19%) | 39 (50%) | 24 (31%) | 0.44:0.56 | 1.4 | 0.8 to 2.6 | 0.15 |
| CE | 13 | 1 (8%) | 9 (69%) | 3 (23%) | 0.42:0.58 | 1.6 | 0.9 to 2.8 | 0.09 |
| Other | 57 | 15 (26%) | 28 (50%) | 13 (24%) | 0.52:0.48 | 1.08 | 0.6 to 1.8 | 0.77 |

**Abbreviations:** IS; Ischemic stroke, LVD; large vessel stroke, SVD; small vessel stroke, CE; cardio-embolic stroke, others; unknown aetiology and other determined aetiology; OR, odds ratio; CI, confidence interval

Values in parantheses are percentages; analysis of allelic association was performed by χ^2^ test

**Table S2.** Stratified analysis by hypertension and age for association of polymorphism at **Arg16Gly (SNP 46 A>G)** position of beta-2 adrenergic receptor gene with ischemic stroke and its subtypes

| **Study group** | **Proportion of Risk allele**  **Case: control** | **Model** | **OR** | **95% CI** | ***P* Value** |
| --- | --- | --- | --- | --- | --- |
| **Hypertensive** | | | | | |
| Ischemic  n= 140 | 0.51:0.46 | Recessive | 1.4 | 0.69 to 2.5 | 0.23 |
|  |  | Dominant | 1.16 | 0.67 to 2 | 0.58 |
| LVD, n=44 | 0.51:0.46 | Recessive | 1.08 | 0.44 to 2.6 | 0.86 |
|  |  | Dominant | 0.89 | 0.37 to 2.1 | 0.79 |
| SVD, n=55 | 0.50:0.46 | Recessive | 1.3 | 0.62 to 2.8 | 0.45 |
|  |  | Dominant | 1.2 | 0.59 to 2.7 | 0.52 |
| **Non-hypertensive** | | | | | |
| IS, n=84 | 0.56:0.46 | Recessive | **2.1** | **1.01 to 4.3** | **0.04** |
|  |  | Dominant | 1.3 | 0.72 to 2.6 | 0.32 |
| LVD, n= 32 | 0.55:0.46 | Recessive | 1.01 | 0.36 to 2.8 | 0.97 |
|  |  | Dominant | 2.6 | 0.83 to 2.3 | 0.01 |
| SVD, n=23 | 0.69:0.49 | Recessive | **3** | **1.09 to 8.5** | **0.03** |
|  |  | Dominant | 8 | 0.99 to 65 | 0.05 |
| **Young onset (Age < 50)** | | | | | |
| IS, n =99 | 0.54:0.46 | Recessive | 1.1 | 0.57 to 2.3 | 0.69 |
|  |  | Dominant | **2.1** | **1.1 to 4.8** | **0.02** |
| LVD, n=29 | 0.51:0.46 | Recessive | 0.96 | 0.3 to 3 | 0.94 |
|  |  | Dominant | 2 | 0.62 to 7 | 0.23 |
| SVD, n=37 | 0.59:0.46 | Recessive | *1.7* | *0.70 to 4.3* | *0.22* |
|  |  | Dominant | ***3.3*** | ***1.06 to 10*** | ***0.03*** |
| **Old onset (Age>50)** | | | | | |
| IS, n=125 | 0.51:0.46 | Recessive | 1.7 | 0.91 to 3.4 | 0.08 |
|  |  | Dominant | 1.4 | 0.55 to 1.9 | 0.88 |
| LVD n=47 | 0.58:0.46 | Recessive | 0.73 | 0.2 to 1.8 | 0.5 |
|  |  | Dominant | 1 | 0.38 to 2.5 | 0.99 |
| SVD, n=41 | 0.52:0.46 | Recessive | 0.67 | 0.27 to 1.6 | 0.39 |
|  |  | Dominant | 1.2 | 0.48 to 3.1 | 0.64 |

**Abbreviations**: IS, Ischemic stroke; OR, odds ratio, CI, confidence interval, LVD, large vessel stroke; SVD, small vessel stroke; Odds ratio estimates based on a multivariate logistic regression model in which demographic and risk factors variables (age, sex, sedentary life style, low education, low economic status, diabetes, dyslipidemia, family history of stroke, high BMI, and smoking) were adjusted, In age-stratified analysis hypertension was also adjusted along with these variables.

**Table S3.** Distribution of genotypic and allelic frequencies of polymorphism at **Gln27Glu (SNP 79 C>G)** position of beta-2 adrenergic receptor gene in controls and ischemic stroke and its subtypes

| **Group** | **No** | **Genotypes** | | | **(Allele) Glu vs. Gln** | | | | |
| --- | --- | --- | --- | --- | --- | --- | --- | --- | --- |
|  |  | Gln/Gln | Gln/Glu | Glu/Glu | Gln:Glu | OR | | CI | *P* Value |
| Con | 224 | 123 (55%) | 88 (39%) | 13 (6%) | 0.75:0.25 |  | | | |
| IS | 224 | 98 (44%) | 91 (40%) | 35 (16%) | 0.64:0.36 | 1.6 | 0.91 to 3.1 | | 0.09 |
| LVD | 76 | 35 (47%) | 27 (35%) | 14 (18%) | 0.64:0.36 | 1.6 | 0.91 to 3.1 | | 0.09 |
| SVD | 78 | 33 (42%) | 35 (45%) | 10 (13%) | 0.65:0.35 | 1.6 | 0.87 to 2.9 | | 0.12 |
| CE | 13 | 6 (46%) | 5 (39%) | 2 (15%) | 0.65:0.35 | 1.6 | 0.87 to 2.9 | | 0.12 |
| Other | 57 | 25 (43%) | 23 (41%) | 9 (16%) | 0.63:0.37 | 1.7 | 0.95 to 3.2 | | 0.06 |

**Abbreviations:** Con, controls; IS, ischemic stroke; LVD, large vessel stroke; SVD, small vessel stroke, CE, cardio-embolic stroke; Others, unknown aetiology and other determined aetiology; Allelic differences were compared by χ^2^ tests

**Table S4.** Analysis stratified by hypertension and age for association of polymorphism at **Gln27Glu (SNP79C>G)** position of beta-2 adrenergic receptor gene with ischemic stroke and its subtypes

| **Study group** | **Risk allele Case:control** | **Model** | **OR** | **95% CI** | ***P* Value** |
| --- | --- | --- | --- | --- | --- |
| **Hypertensive** | | | | | |
| Ischemic  n= 140 | 0.35:0.25 | Recessive | **2.3** | **1.03 to 5.2** | **0.04** |
|  |  | Dominant | **1.9** | **1.1 to 3.1** | **0.01** |
| LVD  n=44 | 0.34:0.25 | Recessive | 2.4 | 0.73 to 8.1 | 0.14 |
|  |  | Dominant | 1.6 | 0.74 to 3.5 | 0.22 |
| SVD  n=55 | 0.35:0.25 | Recessive | 2 | 0.63 to 6.3 | 0.23 |
|  |  | Dominant | **2** | **1.02 to 4** | **0.04** |
| **Non-hypertensive** | | | | | |
| Ischemic  n=84 | 0.37:0.25 | Recessive | **2.8** | **1.2 to 6.7** | **0.01** |
|  |  | Dominant | 1.5 | 0.86 to 2,6 | 0.14 |
| LVD  n= 32 | 0.37:0.25 | Recessive | **4** | **1.3 to 12** | **0.01** |
|  |  | Dominant | 1.7 | 0.77 to 4.1 | 0.17 |
| SVD  n=23 | 0.38:0.25 | Recessive | 2.9 | 0.79 to 11 | 2.9 |
|  |  | Dominant | *1.5* | *0.59 to 4* | *0.36* |
| **Young onset (Age < 50)** | | | | | |
| Ischemic n =99 | 0.29:0.25 | Recessive | 1.4 | 0.53 to 3.9 | 0.47 |
|  |  | Dominant | 1.4 | 0.8 to 2.6 | 0.21 |
| LVD  n=29 | 0.23:0.25 | Recessive | *1.3* | *0.27 to 6.7* | *0.70* |
|  |  | Dominant | *0.93* | *0.33 to 2.5* | *0.88* |
| SVD n=37 | 0.28:0.25 | Recessive | 1.5 | 0.39 to 5.7 | 0.55 |
|  |  | Dominant | 1.2 | 0.54 to 2.9 | 0.58 |
| **Old onset (Age>50)** | | | | | |
| Ischemic n=125 | 0.51:0.25 | Recessive | **3.3** | **1.3 to 3.1** | **0.008** |
|  |  | Dominant | **2.1** | **1.2 to 3.8** | **0.007** |
| LVD n=47 | 0.43:0.25 | Recessive | **3.6** | **1.1 to 11** | **0.025** |
|  |  | Dominant | **2.3** | **1.05 to 5.3** | **0.03** |
| SVD, n=41 | 0.41:0.25 | Recessive | **3.8** | **1.1 to 12** | **0.027** |
|  |  | Dominant | **3.1** | **1.3 to 7.3** | **0.01** |

**Abbreviations:** OR, odds ratio; CI, confidence interval; LVD, large vessel stroke; SVD, small vessel stroke;

Odds ratio estimates based on a multivariate logistic regression model in which demographic and risk factors variables (age, sex, sedentary life style, low education, low economic status, diabetes, dyslipidemia, and family history of stroke, high BMI, and smoking) were adjusted for hypertension stratified analysis. For age-stratified analysis hypertension was also adjusted along with these variables.
